# Interference of bacterial cell-to-cell communication: a new concept of antimicrobial chemotherapy breaks antibiotic resistance

**DOI:** 10.3389/fmicb.2013.00114

**Published:** 2013-05-13

**Authors:** Hidetada Hirakawa, Haruyoshi Tomita

**Affiliations:** ^1^Advanced Scientific Research Leaders Development Unit, Gunma UniversityMaebashi, Gunma, Japan; ^2^Department of Bacteriology and Laboratory of Bacterial Drug Resistance, Gunma University, Graduate School of MedicineMaebashi, Gunma, Japan

**Keywords:** quorum sensing, antagonist, inhibitor, antibiotic resistance, virulence control, autoinducer, homoserine lactone

## Abstract

Bacteria use a cell-to-cell communication activity termed “quorum sensing” to coordinate group behaviors in a cell density dependent manner. Quorum sensing influences the expression profile of diverse genes, including antibiotic tolerance and virulence determinants, via specific chemical compounds called “autoinducers”. During quorum sensing, Gram-negative bacteria typically use an acylated homoserine lactone (AHL) called autoinducer 1. Since the first discovery of quorum sensing in a marine bacterium, it has been recognized that more than 100 species possess this mechanism of cell-to-cell communication. In addition to being of interest from a biological standpoint, quorum sensing is a potential target for antimicrobial chemotherapy. This unique concept of antimicrobial control relies on reducing the burden of virulence rather than killing the bacteria. It is believed that this approach will not only suppress the development of antibiotic resistance, but will also improve the treatment of refractory infections triggered by multi-drug resistant pathogens. In this paper, we review and track recent progress in studies on AHL inhibitors/modulators from a biological standpoint. It has been discovered that both natural and synthetic compounds can disrupt quorum sensing by a variety of means, such as jamming signal transduction, inhibition of signal production and break-down and trapping of signal compounds. We also focus on the regulatory elements that attenuate quorum sensing activities and discuss their unique properties. Understanding the biological roles of regulatory elements might be useful in developing inhibitor applications and understanding how quorum sensing is controlled.

## OUTLINE OF “QUORUM SENSING” IN GRAM-NEGATIVE BACTERIA

The development of antibiotics originated with penicillin, and this approach to the treatment of bacterial infection has been an enormous success. However, the widespread use of antibiotics has resulted in bacteria acquiring resistance in addition to their innate tolerance derived from mechanisms such as biofilm formation and drug efflux. Since the discovery of bacterial quorum sensing in a marine bacterium 40 years-ago, similar systems have been discovered in many organisms, including animal and plant pathogens, and these systems have been characterized along with virulence and drug tolerance determinants. Thus, quorum sensing is now regarded as a potential target for the development of antibacterial agents. In the last 20 years, various quorum sensing inhibitors have been isolated and characterized from natural and chemically synthesized libraries. Some animal and plant infection models have demonstrated the antibacterial efficacy of these agents against quorum sensing pathogens. In this paper, we focus on quorum sensing inhibitors as a novel type of antibacterial agent and also provide an update on recent progress in quorum sensing studies. In the first section, we will review the background and literature relating to bacterial cell-to-cell communication, which is currently termed “quorum sensing.”

### DISCOVERY AND HISTORY

Bacteria are single cell organisms, however, they conduct a bacterial “cell-to-cell” communication activity with the same and/or different species via diffusible chemical compounds, and exhibit group behaviors similar to eukaryotic cells. This concept of social activity between bacteria has been termed “sociomicrobiology” (Parsek and Greenberg, 2005).

Sociomicrobiology was first described in a study carried out in the early 1970s on the bioluminescence phenomenon found in *Vibrio fischeri*, a marine bacterium associated with Hawaiian squid, (Nealson et al., 1970). When the bacteria were grown in shake flasks, expression of the luminescence gene (*lux*) was shown to be relatively low during early exponential growth, but was then followed by a rapid increase in expression during the late exponential and early stationary phases. The luminescence gene in exponential phase cultures can be activated by the addition of cell-free fluid extracts from stationary phase cultures. These observations implied that *Vibrio fischeri* has an environmental sensing system to monitor its own population density, and a signaling substance termed “autoinducer,” which was later shown to be 3-oxohexanoyl-homoserine lactone, activates *lux* expression in high cell density cultures (Eberhard et al., 1981). Currently, over 100 species of bacteria are known to produce autoinducer molecules in a cell density dependent manner similar to *Vibrio fischeri* and this signaling mechanism is now termed “quorum sensing” (Fuqua et al., 1994). Bacteria use three classes of autoinducer for quorum sensing. Acyl-homoserine lactone (AHL) is the most common class of autoinducer used by Gram-negative bacteria, whereas oligopeptide is the major class of autoinducer in Gram-positive bacteria (Dunny and Leonard, 1997). Most of these signals are highly specific and are produced and recognized by a single species. The other class of autoinducer is a 4,5-dihydroxy-2,3-pentanedione (DPD) derivative termed autoinducer-2 (AI-2; Bassler, 2002). It has been suggested that AI-2 is a non-species specific signal which mediates interspecies communication among Gram-negative and Gram-positive bacteria. Although the activity of AI-2 signals has been demonstrated in over 100 species of bacteria, their structures remain largely unknown. Only a few structures of the AI-2 ligand-receptor complex (from *Vibrio harveyi*, *Salmonella* Typhimurium, *Sinorhizobium meliloti*, and *Yersinia pestis*) have been described (Chen et al., 2002; Miller et al., 2004; Pereira et al., 2008; Kavanaugh et al., 2011). In this review, we will focus on the AHL quorum sensing mechanism, as that is the most well defined system of the three quorum sensing families. We will also review progress in the study of effectors which disrupt or attenuate the AHLs-mediated quorum sensing as potential antimicrobial targets.

### ELEMENTS AND REGULATORY SYSTEM OF AHLS QUORUM SENSING

The first AHL molecule to be described was 3-oxohexanoyl-homoserine lactone (typically abbreviated 3-oxo-C_6_-HSL) from *Vibrio fischeri* (Eberhard et al., 1981). Two elements, a signal generator LuxI and the cognate receptor LuxR, regulate the quorum sensing mediated by 3-oxo-C_6_-HSL in this bacterium. The 3-oxo-C_6_-HSL is biosynthesized in a catalytic reaction mediated by LuxI (Engebrecht and Silverman, 1984). The molecule can diffuse into and out of cells, and once a threshold concentration is reached, the 3-oxo-C_6_-HSL binds the cognate receptor, LuxR (Kaplan and Greenberg, 1985; Hanzelka and Greenberg, 1995). This results in a conformational change in LuxR which leads to the activation of the luciferase genes (*lux*) by binding to a specific DNA sequence on the gene promoter.

**FIGURE 1:**
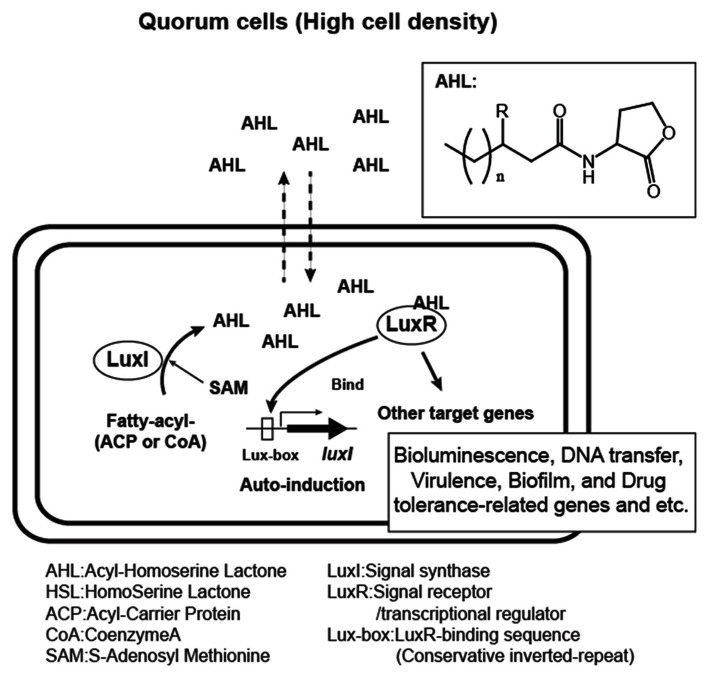
**Acyl-homoserine lactone (AHL) quorum sensing system in Gram-negative bacteria.** Bacteria over a certain threshold of the cell-populations (quorum cells) produce AHL auto-inducers with variations in side chain (*n* = 1, 2, 3…, *R* = H, O, or OH). AHLs are made from fatty acyl substrates modified by ACP (acyl-carrier protein) or CoA (Coenzyme A) and SAM (*S*-adenosyl-methionine) in LuxI signal synthase-mediated reaction and sensed by the cognate LuxR-family receptor.

Since the discovery of the *luxIR* genes, homologous genes have been identified in more than 100 species of Gram-negative bacteria and in some species their quorum sensing activities have also been demonstrated. AHL molecules contain a common homoserine lactone moiety from *S*-adenosyl-methionine (abbreviated SAM) and a specific fatty acid side chain from the bacterial cellular pool. The side chain varies within different species. Therefore, the specificity for AHL signals is conferred by the length and modifications to the fatty acyl groups. Fatty acyl groups are usually 4–18 carbons in length, and some are modified by a 3-oxo or 3-hydroxy substituent, a terminal methyl branch or various degrees of unsaturation (**Figure 1**.). Recently, a new category of homoserine lactone signals that have non-fatty acid side chain substrates has been reported. They utilize phenyl-carbonic acids derived from plant metabolites, or a branched amino acid generated in the process of bacterial amino acid biosynthesis (Schaefer et al., 2008; Ahlgren et al., 2011; Lindemann et al., 2011).

The signal generator LuxI family protein is an enzyme catalyzing the generation of homoserine lactone molecules from SAM and a specific acyl group. However, this enzyme uses the acyl-carrier protein (ACP)-modified thioester form of carbonic acids as a side chain substrate rather than their free form (Schaefer et al., 1996; Parsek et al., 1999). They also have a low affinity for Coenzyme A (CoA)-modified carbonyl substrates.

The LuxR family protein has dual roles as an AHL receptor and a transcriptional regulator. The protein interacts with specific AHLs at an N-terminal signal receiver domain and forms hydrogen bonds between amino acids in the protein and the AHL molecule, which is then subjected to a conformational change in the C-terminal helix-turn-helix (HTH) domain, which enables it to bind to a conserved inverted repeat DNA sequence termed the “Lux-box” located upstream of the target gene’s promoter. LuxR family proteins usually respond to AHLs produced by the corresponding synthase, however, there are some proteins that have a wide range of AHL-binding specificity. For example, CviR, a LuxR homolog from *Chromobacterium violaceum* is able to respond to AHLs with side chains that are C_4_ to C_8_ in length (Mcclean et al., 1997).

Quorum sensing is known to control a variety of bacterial genes involved in bioluminescence, plasmid transfer, virulence, the biosynthesis of secondary metabolites and antibiotics, and biofilm formation. Comprehensive transcriptome and computational promoter analyses have revealed quorum sensing-controlled genes in several organisms. For example, in *Vibrio fischeri*, only (0.6% of total) genes are controlled by LuxR-3-oxo-C_6_-HSL (Antunes et al., 2007), whereas more than 300 genes (6% of total) are regulated during quorum sensing in the opportunistic pathogen *Pseudomonas aeruginosa* (Schuster et al., 2003).

## QUORUM SENSING AND BACTERIAL INFECTIOUS DISEASES

Although quorum sensing was originally discovered in a bioluminescence study using a marine organism, extensive studies in this area have been performed with pathogenic bacteria. There is increasing evidence that bacteria use the quorum sensing mechanism to regulate their own virulence genes. Quorum sensing is considered to be a strategic tool enabling bacteria to accomplish their infection processes and survive in the host. The physiological benefit allows the bacterial cells to multiply without displaying overt virulent behavior until a certain threshold population density is reached. As a consequence, a coordinated immunological response by the host is only made when the bacterial population is high, which increases the likelihood that any defenses will be successfully overwhelmed, thereby enhancing the survival prospects of the bacteria. In this section, we will summarize the studies that have investigated the contribution of quorum sensing to bacterial virulence and infectious disease in *Pseudomonas aeruginosa*, which is the quorum sensing pathogen studied in the most detail.

*Pseudomonas aeruginosa* is an opportunistic pathogen, is commonly associated with nosocomial infections and is infectious in immune-compromised patients. This organism is also known for the chronic infection it causes in individuals with the genetic disease cystic fibrosis (CF) which can result in respiratory failure. In addition, this bacterium is regarded as a “model organism” in the quorum sensing field. The AHL signals produced by *Pseudomonas aeruginosa* are 3-oxododecanoyl-homoserine lactone (3-oxo-C_12_-HSL; Pearson et al., 1994) and butanoyl-homoserine lactone (C_4_-HSL) (Pearson et al., 1995). They are generated by AHL synthases called LasI and RhlI and subsequently bind to the cognate receptors called LasR and RhlR, respectively. This dual quorum sensing system is hierarchical. When the bacterial cell density reaches a particular threshold, the LasIR quorum sensing system is initiated. The 3-oxo-C_12_-HSL-LasR complex activates *rhlI* expression as well as LasR-controlled genes including *lasI*, the cognate signal synthase, which then leads to activation of the RhlIR system. Either or both the *las* and *rhl* systems activate the production of virulence factors such as elastase, alkaline protease, exotoxin A, rhamnolipids, pyocyanin, lectines, and superoxide dismutase (Smith and Iglewski, 2003). In addition to LasR and RhlR, there is a third LuxR-family protein named QscR that is a homolog of LasR/RhlR, but does not have the cognate signal synthase (Chugani et al., 2001). QscR can bind with 3-oxo-C_12_-HSL as well as LasR, and also with heterologous C_12_, C_10_, 3-oxo-C_10_, and 3-oxo-C_6_-HSLs (Lee et al., 2006; Oinuma and Greenberg, 2011). Apart from these AHLs, *Pseudomonas aeruginosa* also produces one non-AHL quorum sensing molecule termed “the *Pseudomonas* quinolone signal (PQS)” (Pesci et al., 1999). The PQS is synthesized by PqsABCD and PqsH from anthranilate that is an intermediate in the tryptophan biosynthetic pathway, and responded by PqsR (MvfR), a LysR-like protein. According to some studies, PQS and AHL-quorum sensing (*las* and *rhl*) are interlinked. The production of PQS is activated by *las* system and PQS influences the expression of C_4_-HSL-regulated genes in *rhl*-dependent and –independent manners, suggesting that PQS could be also important for virulence of the organism. Highlight of PQS studies is reviewed elsewhere (Diggle et al., 2006; Huse and Whiteley, 2011)

Infection studies with mice have demonstrated the contribution of quorum sensing in the pathogenesis of *Pseudomonas aeruginosa*. *Pseudomonas* strains with mutations in quorum sensing-regulated genes induce less tissue destruction and pneumonia and result in lower mortality compared with the wild-type (Rahme et al., 1995; Tang et al., 1996; Rumbaugh et al., 1999, 2009). Additional studies using alternative infection models with *Caenorhabditis elegans*, *Arabidopsis thaliana*, and *Dictyostelium discoideum* also have illustrated decreases in virulence with quorum sensing mutants (Rahme et al., 1995; Tan et al., 1999; Cosson et al., 2002). These model studies using an acute-infected animal host illustrate the contributions of the quorum sensing system to *Pseudomonas aeruginosa* infections. There are a few reports that *Pseudomonas aeruginosa* quorum sensing is also responsible for chronic lung infections. *Pseudomonas aeruginosa* with mutations of *lasI* and/or *rhlI* showed milder lung infections in mice and rat models (Wu et al., 2001; Imamura et al., 2005). However, the contribution of quorum sensing to chronic infections should be discussed in caution because accumulation of *lasR* mutants has been frequently observed in both many clinical isolates from CF patients and long-term laboratory cultures (Cabrol et al., 2003; Diggle et al., 2007). The *lasR* variants are considered social cheaters (Sandoz et al., 2007; Dandekar et al., 2012). LasR activates genes encoding for extracellular proteases which undertake proteolyses to cooperatively crop common metabolic/energy substrates, reasonably, the LasR-mediated quorum sensing can be promoted under nutrient-limited conditions in the presence of the particular protease substrates. The *lasR* cheaters exploit the social benefit provided by the cooperators (*lasR*-intact parent strain) saving biological costs to conduct their quorum sensing. It might be critical to answer about total quorum sensing activity of the bacterial population in infection sites and frequency of the mutant appearance.

Moreover, quorum sensing has also been shown to influence biofilm development. Biofilm is a biological architecture of aggregated microbes on a surface. It is closely associated with virulence because biofilm cells embedded within an extracellular matrix are less susceptible to antibacterial reagents than free floating cells (Nickel et al., 1985; Mah and O’Toole, 2001; Drenkard, 2003). As a result, biofilm infections tend to be chronic and difficult to eradicate. *Pseudomonas aeruginosa* is the principal pathogen in the lungs of patients with CF. The bacterium is known to exist there as a biofilm and produce significant amounts of quorum sensing molecules (Singh et al., 2000). There is a report that the *Pseudomonas aeruginosa* 3-oxo-C_12_-HSL signal is involved in the maturation of a biofilm. A *lasI* mutant formed immature biofilms that, unlike wild-type biofilms, were sensitive to sodium dodecyl sulfate (SDS; Davies et al., 1998). Another report predicted that the *rhl* system is also important for biofilm development. The *rhl* defective *Pseudomonas aeruginosa* does not produce the rhamnolipid surfactants that are important for maintaining the biofilm architecture in the later stages (Davey et al., 2003). However, it should be noted that there is some conflict of opinion and confusion regarding the degree of involvement of quorum sensing in biofilm regulation. Biofilm architecture is easily influenced by growth conditions and wild-type, and the quorum sensing mutants might form identical biofilm under certain conditions (See reviews; Parsek and Greenberg, 2005; Parsek and Tolker-Nielsen, 2008). A number of animal infection studies carried out with different objectives have shown that quorum sensing is required for virulence, therefore in the context of virulence, it is generally believed that quorum sensing contributes to the formation of a functional biofilm. Studies of biofilm dynamics to define complexity and trace the development process have being developed over the last 10 years and these will provide insights into the actual role of quorum sensing on biofilm biology. In addition to biofilm development, quorum sensing induces the expression of the drug efflux system MexA-MexB-OprM and confers tolerance to a wide range of antimicrobial agents by extruding them from the cytoplasm (Poole, 2001; Maseda et al., 2004). Thus, quorum sensing is a key factor in determining the success of infection in host animals for *Pseudomonas aeruginosa*

## QUORUM SENSING INTERFERENCE

**FIGURE 2:**
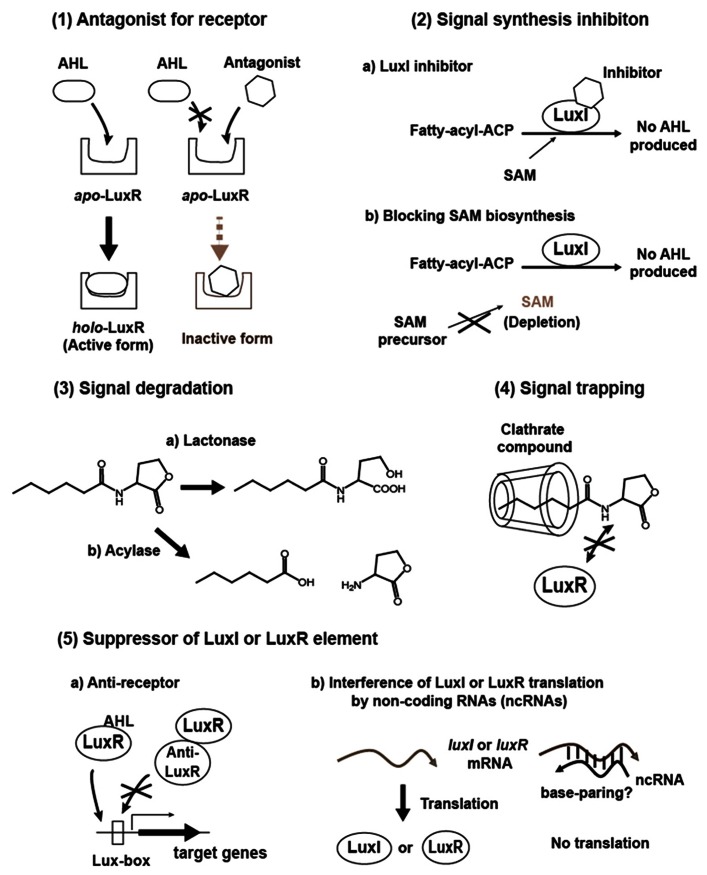
**Proposed methods for AHL quorum sensing interference**.

Interfering with quorum sensing is expected to become a powerful strategy to control virulence and antibiotic tolerance in quorum sensing pathogens, and can be applied within antimicrobial chemotherapy to overcome bacterial infections. To date, methods that can be used to disrupt quorum sensing include (1) Antagonizing signal binding to LuxR-family receptor, (2) Inhibition of signal production, (3) Degrading signals, (4) Trapping signals, and (5) Suppression of synthase and receptor activities, stabilities or productions (**Figure 2**). A brief background and recent progress in these studies will be given in the following section.

### ANTAGONIST FOR LUXR-FAMILY RECEPTOR

**FIGURE 3:**
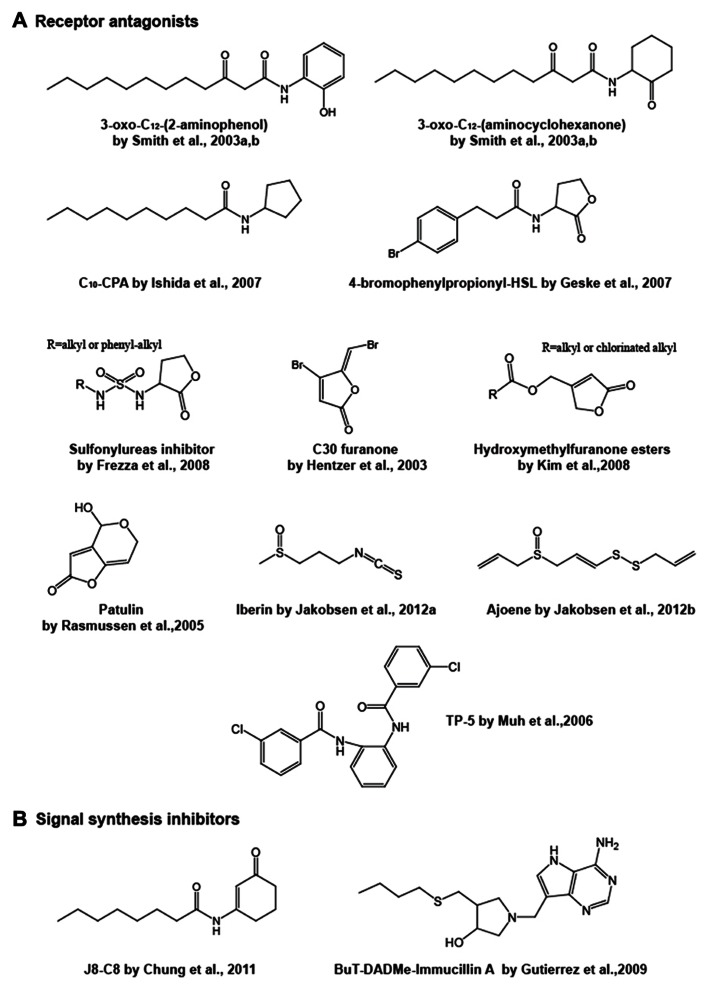
**Chemical structures of representative receptor antagonists (A)** and signal synthesis inhibitors **(B)**.

The initial step in quorum sensing is to bind a specific AHL signal to a LuxR protein. Thus, antagonists that interfere with the AHL-the receptor binding are potential quorum sensing inhibitors. Various natural and synthetic compounds have been tested for their antagonistic activity (Chemical structures of representative inhibitors are drawn in **Figure 3**.). In general, analogs are potential antagonists of the native AHL signal. In three early studies, analogs with alternations in the acyl side chain of 3-oxo-C_6_-HSL for *Vibrio fischeri*, 3-oxo-C_12_-HSL for *Pseudomonas aeruginosa* and** 3-oxo-C_8_-HSL for *Agrobacterium tumefaciens* were demonstrated to inhibit the binding of native AHLs (Passador et al., 1996; Schaefer et al., 1996; Zhu et al., 1998). These studies focused particularly on the length of the acyl side chains. These cognate receptors are able to bind some analogs at a higher affinity than native AHL ligands, but the analogs then inactivate gene expression, thus they are antagonists.

Following earlier studies, a library of synthetic analogs to the *Pseudomonas aeruginosa* *las* quorum sensing molecule 3-oxo-C_12_-HSL was constructed by Smith et al. (2003a,b). The homoserine lactone moiety of their compounds was replaced with different alcohols, amines and/or a 5- or 6-membered ring. In their high throughput screening using a *lasI* promoter-fused *gfp* reporter strain, three compounds acted as antagonistics against 3-oxo-C_12_-HSL-LasR-mediated quorum sensing. 3-Oxo-C_12_-(2-aminophenol) and 3-oxo-C_12_-(aminocyclopentanol) are able to inhibit the LasR activation attributed to the 3-oxo-C_12_-HSL-LasR interaction. On the other hand, 3-oxo-C_12_-(aminocyclohexanone) appeared to target not only LasR but also RhlR, which is the second quorum sensing receptor in *Pseudomonas aeruginosa*, although this antagonist still has a dodecanoyl (C_12_)-side chain. A synthetic analog of C_4_-HSL, *N*-decanoyl cyclopentyl-amide (abbreviated C_10_-CPA), has also been found to target both LasR and RhlR proteins (Ishida et al., 2007). C_10_-CPA inhibits *lasB* and *rhlA* gene activation by 3-oxo-C_12_-HSL and C_4_-HSL with IC50 (half-maximal inhibition) of 80–90 μM, respectively, and inhibition results in reductions in elastase, pyocyanin, and rhamnolipid levels and biofilm formation. Most of these analog compounds are modified with a homoserine lactone moiety and/or a side chain. Changes in the amide function bridging the lactone ring and the fatty acid also influences the AHL binding activity to receptor proteins, since the amide forms hydrogen bonds with a conserved tyrosine and aspartic acid in the AHL binding pocket (Vannini et al., 2002; Zhang et al., 2002). Changing the amide to a sulfonamide and/or a urea has been predicted to result in the formation of an additional hydrogen bond between a tyrosine residue in the ligand pocket and the sulfonamide, and to strengthen the hydrogen bond between the aspartic acid and the external NH of urea (Castang et al., 2004; Frezza et al., 2006, 2008). Compounds with either or both modifications have showed antagonistic behaviors with the *Vibrio fischeri* LuxR receptor. In recent studies, selective and broad-spectrum antagonists active across multiple species have also been developed. C_8_-HSL, C_10_-HSL, 4-bromophenylpropionyl-HSL and 4-iodophenylacetyl-HSL simultaneously antagonize the AHL-bindings to the receptor proteins of TraR in *Agrobacterium tumefaciens*, LuxR in *Vibrio fischeri* and LasR in *Pseudomonas aeruginosa*, while several other analogs work on two of these species (Geske et al., 2007).

Some natural compounds have also been demonstrated to behave as antagonists. Halogenated acyl-furanones, which are structurally similar to AHLs, and are derived from the marine algae *Delisea pulchra* are one of the most-studied antagonist groups (Givskov et al., 1996). These naturally occurring compounds displace the 3-oxo-C_6_-HSL signal from its cognate LuxR receptor protein, thus inhibiting the quorum sensing-mediated gene expression (Manefield et al., 1999). The SwaR receptor for C_4_-HSL in *Serratia liquefaciens* is also a target for the antagonists, and the failure of C_4_-HSL-SwaR interaction results in a reduction in the swarming motility linked to surface colonization and biofilm formation (Rasmussen et al., 2000). Gram et al. (1996) have reported that a furanone isolated from the furanones pool of secondary metabolites produced by *Delisea pulchra* inhibits swarming motility in *Proteus mirabilis* without affecting cell growth and swimming motility, whereas other furanones have no inhibitory activity. However, the regulatory target in this system is not known. Although natural furanones have a limited inhibitory effect on the quorum sensing of *Pseudomonas aeruginosa*, the synthetic analogs “C30” and “C56,” which lack the alkyl side chain, exhibit interference in the LasR-mediated *Pseudomonas aeruginosa* *lasB* gene** expression (encoding the elastase that is associated with the virulence), increase the susceptibility to antimicrobial agents on biofilm cells, promote the bacterial clearance on the lung of infected mice and prolong the survival time of the mice (Hentzer et al., 2002, 2003; Wu et al., 2004; Christensen et al., 2012). Taha et al. (2006) have discovered three LasR antagonists by *in silico* screening with pharmacophore modeling utilizing the authentic furanone inhibitors C-30 and C-56 as leading compounds. These are phenyl compounds incorporating a mercury or lead atom bound by covalent bonds and have been shown to inhibit quorum sensing-driven pyocyanin and pyoverdin production. Subsequent studies have developed other furanone derivatives based on natural furanone core structures. A series of hydroxymethylfuranone esters condensed with fatty acids that have a modified carbon length or are chlorinated at the terminal has been synthesized (Kim et al., 2008). These compounds have been shown to repress the LasR-driven reporter expression in a *lasI*-*lacZ* fusion by competing with exogenous 3-oxo-C_12_-HSL binding to a recombinant LasR in *Escherichia coli*, and they also inhibit biofilm formation on the flow cell system in *Pseudomonas aeruginosa*. The authors have built the inhibitor-LasR protein docking models. According to the *in silico* modeling analyses, the inhibitors are predicted to bind preferentially to the receptor rather than the natural ligand, but fail to change the conformation of LasR to the “active” form, which suggests they have antagonistic activities. In the same year, another group reported a series of furanone antagonists whose structures are closer to AHL. They replaced the homoserine lactone moiety of AHL with a furanone. These molecules dock with the LuxR protein at its binding pocket (Estephane et al., 2008). However, we should note a report that halogenated furanones destabilize LuxR receptors rather than antagonizing (Manefield et al., 2002). Therefore, as for exact action mode of this type of inhibitors, there is still some confusion. Remarkably, a very recent study has highlighted the possibility that furanone resistance might arise in *Pseudomonas aeruginosa* (Maeda et al., 2012). Maeda et al. (2012) have observed that a mutation in the *mexR* gene in *Pseudomonas aeruginosa* decreases its susceptibility to the synthetic C-30 furanone. As the *mexR* gene product functions as a repressor for the *mexAB*-*oprM* operon encoding a multidrug efflux system, the inactivation of *mexR* gene presumably leads to overexpression of MexAB-OprM, thereby enhancing the efflux of the furanone inhibitor.

In addition, other natural AHL inhibitors have been identified, such as patulin and penicillic acid produced by fungi (Rasmussen et al., 2005), iberin from horseradish extracts (Jakobsen et al., 2012a) and ajoene from garlic (Jakobsen et al., 2012b). These natural products inhibit *Pseudomonas aeruginosa* quorum sensing of either or both the *las* and *rhl* systems. Patulin and penicillic acid have structures reminiscent of the furanone compounds originally discovered in *Delisea pulchra,* whereas iberin and ajoene are structurally unrelated linear sulfide compounds, but can compete with *Pseudomonas aeruginosa* AHLs for binding to LasR or RhlR.

Aside from AHL structural analogs, a research group has identified three inhibitors for the LasR protein in combination studies by screening a library of 200,000 compounds and *in silico* structure modeling. A tetrazole and a phenyl ring compound with a common 12-carbon alkyl tail designated “PD12” and “V-06-018”, respectively, were isolated from the library (Muh et al., 2006b). Both compounds inhibited LasR-controlled gene expression and expression of the virulence factors elastase and pyocyanin. A triphenyl compound designated “TP-5” was originally predicted to be an antagonist by the LasR-ligand docking model built from the 3D-structure of the ligand-bound TraR protein in *Agrobacterium tumefaciens* (Vannini et al., 2002; Zhang et al., 2002), which is highly homologous to LasR. Interestingly, it is more likely that TP-5 interacts with LasR by forming a hydrogen bond with Asp-73 of the polypeptide and exhibits LasR-inhibitory activity despite being structurally unrelated to the natural ligand. This compound was derived from a parent compound designated “TP-1” from their library of compounds. TP-1 mimics 3-oxo-C_12_-HSL activity, and therefore acts as an agonist for LasR (Muh et al., 2006a).

Information on the 3D-structures of the LuxR-family receptors has enabled us to discover a variety of antagonists. However, this information is not enough. We need to know how the conformational change in the receptor occurs to distinguish between a “true ligand” (agonist) and “fake ligand” (antagonist). The crystal structure only provides a snapshot of the information required. Although the structures of receptor-AHL binding form are available for a few species, we need to obtain both structures; an antagonist-binding form and a ligand-free form. Recently, the crystal structure of CviR from *Chromobacterium violaceum* with an antagonist has been reported and so far this is the only example (Chen et al., 2011). On the other hand, analysis of the signal-free form is currently a difficult task because LuxR-family proteins are considered to be unstable in the absence of the cognate signal, and are eventually subjected to proteolysis (Zhu and Winans, 2001). However, although a major challenge, it is important to gain information about these structures.

### SIGNAL SYNTHESIS INHIBITION

Interfering with AHL synthesis is another straightforward approach to the inhibition of quorum sensing. Simply, if no AHL is produced, no quorum sensing occurs. Early studies on quorum sensing inhibitors have focused on antagonists of AHL receptors. However, there have been few studies on inhibitors of AHL synthesis and data is very limited. In one of these studies, it was found that several analogs of SAM, which is the second substrate for LuxI synthases, inhibit the LuxI reaction (Parsek et al., 1999). However, two breakthrough studies have been recently carried out. The first study identified a C_8_-HSL analog that binds to AHL synthase, thereby inhibiting its enzymatic activity (Chung et al., 2011). The second study described compounds targeting the reaction activity of 5^′^-methylthioadenosine nucleosidase (MTAN, alternatively abbreviated “Pfs”) involved in SAM recycling (Gutierrez et al., 2009).

The compound in the first study is *N*-(3-oxocyclohex-1-enyl)octanamide (named J8-C8) (**Figure 3**), which was isolated from Smith’s library (Smith et al., 2003a) and was characterized as an inhibitor for TofI, a C_8_-HSL generator of the LuxI-family protein in *Burkholderia glumae* (Chung et al., 2011). J8-C8 significantly inhibited C_8_-HSL production by TofI in a dose dependent manner, furthermore the effect was enhanced in the presence of MTA. Generally, the LuxI-family protein synthesizes a specific AHL from an acylated ACP and SAM, eventually AHL is released with holo-ACP and 5^′^-methylthioadenosine (MTA) as by-products from the protein. In a TofI/J8-C8/MTA ternary crystal structure, J8-C8 binds to TofI occupying the binding site for the acyl chain of the cognate substrate C_8_-ACP. Simultaneously, a second substrate (MTA) binds to the binding site for SAM, which accounts for the synergistic effect of MTA.

The latter study describes AHL synthesis inhibitors that are transition state analogs of MTAN. The MTAN enzyme catalyses the hydrolytic deadenylation of MTA and *S*-adenosyl homocysteine (SAH) and produces 5^′^-methylthioribose (MTR) and *S*-ribosylhomocysteine (SRH) which are steps in SAM biosynthesis. MTAN enables AHL-producing bacteria to recycle SAM from the MTA released as a by-product after AHL syntheses. In addition, SRH also becomes a precursor for AI-2 generation (Xavier and Bassler, 2003). Thus, the MTAN inhibition provides a method of blocking not only AHL production, but also AI-2. Three analogs of the transition state during the reaction from the MTA substrate into MTR and adenine, have been designed and are named 5^′^-methylthio- (MT-), 5^′^-ethylthio- (EtT-), and 5^′^-butylthio- (BuT-) DADMe-ImmucillinAs (**Figure 3**; Gutierrez et al., 2009). According to a 3D-structural analysis of MTAN in *Vibrio cholerae* with BuT-DADMe-ImmucillinA, the inhibitor binds to the catalytic active site of the protein producing hydrophobic stacking interactions. These analogs, including BuT-DADMe-ImmucillinA, have been shown to inhibit MTAN activity with IC50 values at the nM level and reduce AI-2 production and biofilm formation in *Vibrio cholerae* and *Escherichia coli* O157. Although this study principally described the AI-2 effect, the researchers might investigate AHL quorum sensing in the near future.

### DEGRADATION ENZYMES

In addition to small molecules which interfere with signal sensing or generation, signal breakdown by catalytic enzymes is an alternative strategy. Two classes of enzymes, lactonase and acylase, are known to perform this function. The former is a catalytic enzyme that cleaves the homoserine lactone ring and the latter catalyzes the hydrolysis of an amide bond between the homoserine lactone moiety and a fatty-acyl group. The degraded AHL products are no longer active in quorum sensing, therefore the phenomenon is often called “quorum quenching.” A lactonase was originally identified and purified from a Gram-positive *Bacillus* strain and the enzyme was designated “AiiA,” meaning autoinducer inactivation. The protein sequence has no significant similarity to any known sequences, but contains a HXHXDH zinc-binding motif that is conserved in glyoxalase II, metallo β-lactamase and arylsulfatase (Dong et al., 2000). The purified AiiA protein cleaves the homoserine lactone ring in C_4_ to C_12_-HSLs, with or without substitution at carbon three position including 3-oxo-C_6_-HSL produced by a plant pathogen, *Erwinia carotovora*. Heterologous expression of the *aiiA* gene in *Erwinia carotovora* resulted in a remarkable decrease in quorum sensing-activating gene expression and less virulence to plants. Also, *aiiA*-expressing transgenic tobacco and potato were tolerant to the bacterial infection (Dong et al., 2001). There are similar bioengineering studies utilizing AiiA lactonase. For example, AiiA overexpression in *Pseudomonas aeruginosa* and *B. thailandensis* impaired their quorum sensing activities through the degradation of signals (Reimmann et al., 2002; Ulrich, 2004). Following these AiiA studies, *aiiA* homologs genes have been identified from other species such as *Agrobacterium tumefaciens* (Zhang et al., 2002) and *Arthrobacter* sp., (Park et al., 2003) and their enzymatic activities have been demonstrated. In addition, a subclass of AHL lactonases has been recently discovered in a species of soil bacterium. Unlike AiiA, they have no conserved HXHXDH zinc-binding motif. For example, QsdA in *Rhodococcus erythropolis* is a phosphotriesterase (PTE)-like protein that has other zinc-binding domains instead of the HXHXDH motif and can degrade AHLs which have an acyl chain of C_6_–C_14_ in length (Uroz et al., 2008). However, unlike typical PTE enzymes, the protein is unable to cleave the phosphotriester bond. AiiM in *Microbacterium testaceum* isolated from a potato leaf (Wang et al., 2010) and three BpiB isomer proteins from soil metagenomic clones (Schipper et al., 2009) do not have any putative zinc-binding domains. AiiM has been deduced to belong to alpha/beta hydrolase fold family. The protein prefers C_6_ to C_12_-HSLs with 3-oxo substitution to those without substitution as degradation substrates. The expression of AiiM in the plant pathogen *Pectobacterium carotovorum* subsp. *carotovorum,* which is a 3-oxo-C_6_-HSL producer, reduced virulence against the potato tissue (Wang et al., 2010). The series of BpiB proteins were originally isolated from soil-derived metagenomic libraries with a *traI*-*lacZ* *Agrobacterium tumefaciens* reporter strain that can respond to 3-oxo-C_8_-HSL, then protein expressing clones which attenuated *traI*-*lacZ* activity were isolated. Two out of the three BpiB proteins, designated BpiB01 and BpiB04, show no similarity to any known proteins while the other protein, BpiB07 shares some sequence similarity with the esterase-lipase superfamily proteins. In addition to the degradation of 3-oxo-C_8_-HSL, all the clones inhibited *Pseudomonas aeruginosa* swarming motility and biofilm formation controlled by 3-oxo-C_12_-HSL and C_4_-HSL (Schipper et al., 2009). AHL lactonase was originally characterized as a zinc-binding protein and it has been shown that mutations in the HXHXDH zinc-binding domain in some AiiA family lactonases result in them losing their function. However, based on their amino acid sequences, it is still unclear whether AiiM and BpiBs bind zinc. Recently, a new type of AHL lactonase has been isolated from the marine bacterium *Pseudoalteromonas byunsanensis*. The identified ORF appears to encode a hybrid membrane protein that has a GDSL (consensus Gly-Asp-Ser-Leu motif) hydrolase domain at the N-terminal of a RND (resistance-nodulation-cell-division)-type multidrug efflux transporter (Huang et al., 2012). The truncated form, including the GDSL hydrolase function, is designated as QsdH and has a catalytic activity for the C_4_ to C_12_-HSLs (with or without 3-oxo substitution) lactonase reaction, and co-inoculation of the plant pathogen *Erwinia carotovora* with a recombinant QsdH-overexpressing *Escherichia coli* has resulted in milder lesions on potato tissues compared to a co-inoculation without QsdH.

In addition to environmental microorganisms, mammalian enzymes also have AHL lactonase activities (Chun et al., 2004). Human has three paraoxygenases (PON1, PON2, and PON3) with a distinct substrate specificity and expression pattern. There are reports that they cleave lactone rings in a series of AHLs (Draganov et al., 2005; Ozer et al., 2005)

The other family of AHL degradation enzymes is AHL acylase. This was first described in *Variovorax paradoxus*, although the gene which is responsible for the reaction has not been yet identified (Leadbetter and Greenberg, 2000; Leadbetter, 2001). The organism was isolated from soil based upon its ability to utilize 3-oxo-C_6_-HSL as both an energy and nitrogen source. Hypothetically, AHLs is initially cleaved into a fatty acid and homoserine lactone moiety by an uncharacterized acylase in first reaction step, and subsequently the fatty acid is subjected to beta-oxidation as an energy material, while the homoserine lactone is degraded into ammonium chloride and carbon dioxide. The first AHL acylase to be characterized is AiiD from *Ralstonia eutropha* (Lin et al., 2003). The polypeptide is most similar to the aculeacin A acylase (AAC) from *Actinoplanes utahensis* and it also shares significant similarities with the cephalosporin and penicillin acylases, which are members of the N-terminal (Ntn) hydrolase superfamily. AiiD has been purified as a glutathione *S*-transferase (GST) fusion protein and its AHLs cleavage spectrum has been investigated. The GST-AiiD protein effectively hydrolyzes an amide bond on AHLs with longer fatty acyl side chains, such as 3-oxo-C_8_-HSL, 3-oxo-C_10_-HSL and 3-oxo-C_12_-HSL whereas it is less active against shorter side chain substrates as 3-oxo-C_6_-HSL. Heterologous AiiD expression in *Pseudomonas aeruginosa* has also been shown to abolish the accumulation of both 3-oxo-C_12_-HSL and C_4_-HSL and the killing of *Caenorhabditis elegans*. Based on the AiiD sequence, homologues have been identified in other organisms. Three acylases in *Pseudomonas aeruginosa* and closely related species, designated PvdQ, QuiP, and PA0305 (alternatively named HacB), respectively, are well-characterized (Huang et al., 2003, 2006; Wahjudi et al., 2011). They participate in the degradation of 3-oxo-C_12_-HSL, but not C_4_-HSL. However, their expression is considered to be highly regulated and turned off in standard experimental conditions (usually aerobically growth at 37 degree in rich medium) because wild-type *Pseudomonas aeruginosa* accumulates a large amount of 3-oxo-C_12_-HSL during early stationary phase. When these acylases are constitutively produced from exogenous plasmids, a significant reduction of 3-oxo-C_12_-HSL accumulation in the medium is observed. Some other homologous AHL acylases have also been identified in *Streptomyces* sp. from soil samples, the fish-associated bacterium *Shewanella* sp. and the nitrogen-fixing cyanobacterium *Anabaena* sp. (Park et al., 2005; Morohoshi et al., 2008; Romero et al., 2008). These studies provide us with not only extensive ideas for quorum sensing inhibitor applications, but also stimulate our general biological interest as to why AHLs-degrading organisms are widespread in nature. Thus far, the physiological benefits of degradation enzymes are presumed to be specific to AHL utilization as a nutrient resource, the detoxification of lactone ring compounds, the jamming of quorum sensing in pathogens as an innate bio-defense mechanism, and the modulation of the quorum sensing activity.

### SIGNAL TRAPPING

An alternative technique for the attenuation of quorum sensing based on the trapping of AHLs has been created. This method arose from the observation that quorum sensing does not occur when the AHL concentration is maintained below a threshold level, thus an AHL interceptor would act as a quorum sensing inhibitor. Cyclodextrins are well known to form stable aqueous complexes with many organic compounds. In an initial study, C_4_-HSL was reported to be an entry substrate for a cyclodextrin donor, and a bacterial culture containing the cyclodextrin suppressed RhlR-activated *rhlA* gene expression in *Pseudomonas aeruginosa* (Ikeda et al., 2002). The C_6_-HSL, C_7_-HSL, C_8_-HSL, and 3-oxo-C_6_-HSL from *Serratia marcescens* and C_4_-HSL from *Pseudomonas aeruginosa* can be trapped and this results in the decrease in the production of the quorum sensing-induced red-pigment (prodigiosin; Kato et al., 2006). Currently, the use of cyclodextrin as a method of quorum sensing interference is still immature, although it is well studied as a cholesterol remover and as a carrier for medical applications such as in Niemann–Pick disease (See review Vance and Peake, 2011). For applications to antibacterial and bio-fouling materials, further technical studies are required, for example, a chemical engineering approach to increase the solubility and the stability of the cyclodextrin-AHL inclusion complex may be successful in the future.

### UNKNOWN MECHANISM: MACROLIDE ANTIBIOTICS AS A CASE OF STUDY

Macrolide antibiotics have been shown to inhibit *Pseudomonas aeruginosa* quorum sensing. Since macrolides are relatively hydrophobic and are large sized-molecules, these antibiotics are generally believed to be ineffective against Gram-negative bacteria due to their low permeability and exclusion from the bacterial cytoplasm (Nikaido and Vaara, 1985; Nikaido, 1996). However, surprisingly, there have been reports from clinical trials showing that long term treatment with macrolide antibiotics at sub MIC eases the chronic lung infectious diseases caused by *Pseudomonas aeruginosa* in patients with CF and diffuse panbronchiolitis (DPB; Keicho and Kudoh, 2002; Southern et al., 2011). A number of mechanisms for the macrolide action on the bacterium and host have been proposed (See reviews, Tateda et al., 2007; Kanoh and Rubin, 2010). One proposal is that the drug influences quorum sensing. Azithromycin, the 15-membered ring macrolide has been shown to repress the activity of *Pseudomonas aeruginosa* quorum sensing based on both the levels of 3-oxo-C_12_-HSL and C_4_-HSL syntheses and the expression of *las* and *rhl*-activated gene/protein such as elastase, rhamnolipid, and pyocyanin (Tateda et al., 2001; Wagner et al., 2005; Nalca et al., 2006; Skindersoe et al., 2008). The azithromycin efficacy at sub-MIC is presumably attributed to the reduction of 3-oxo-C_12_-HSL and C_4_-HSL levels because a subset of genes involving SAM biosynthesis is partly repressed by azithromycin (Kai et al., 2009). Elucidation of its exact molecular action and target is the next question to be answered.

### SUPPRESSORS OF QUORUM SENSING

It is known that some quorum sensing bacteria have regulatory elements which impede their quorum sensing. It is speculated that the physiological implications of an intrinsic modulation mechanism in quorum sensing is a tightly controlled repression of quorum sensing-controlled genes under a threshold population, a delay in quorum sensing initiation, and a slowing of its regulatory circuit or fine-tuning of its activity at a specific level. Apart from their actual roles, it might be possible to apply these suppressors to a quorum sensing inhibitory method, because if we are able to artificially manipulate the function and cellular level of these elements, quorum sensing will be controlled.

Anti-LuxR activators inhibiting quorum sensing activation have been reported in *Agrobacterium tumefaciens* and *Pseudomonas aeruginosa*. TrlR from *Agrobacterium tumefaciens* is a homologue of TraR, an AHL receptor protein, but lacks a DNA binding domain (Chai et al., 2001). The protein forms an inactive heterodimer with TraR. The other anti-TraR proteins, TraM and its homologue TraM2, also interact with TraR to prevent its DNA binding (Fuqua et al., 1995; Hwang et al., 1995; Swiderska et al., 2001; Wang et al., 2006). These mutants confer constitutive AHL signal accumulation even in the absence of octopine, which is a quorum sensing initiator, and also confer hyper-plasmid conjugative transfer efficiency with excessive activation of the quorum sensing. Like anti-TraR in *Agrobacterium tumefaciens*, QslA is an anti-LasR protein in *Pseudomonas aeruginosa* (Seet and Zhang, 2011). The *qslA* null mutant is able to respond to much lower levels of the quorum sensing signal than the parent, resulting in higher quorum sensing activity, such as elevated exo-protease and elastase production. QscR, an orphan LuxR-family protein in *Pseudomonas aeruginosa,* inhibits a number of LasR- RhlR-activated genes by protein–protein interaction with LasR and RhlR, respectively, and suppresses virulence in a *Drosophila* infection model (Chugani et al., 2001; Ledgham et al., 2003). The unique small protein QteE controls the stability of LasR protein in *Pseudomonas aeruginosa,* but affects neither its transcription nor translation (Siehnel et al., 2011). In the absence of the *qteE* gene, LasR is more stable at a low cell density culture and overproduction of QteE reduces the LasR stability. In addition to these anti-activators, RsaL is a repressor and simultaneously binds to the *lasI* promoter with LasR in *Pseudomonas aeruginosa* (Rampioni et al., 2006, 2007). The *rsaL* mutant results in unlimited 3-oxo-C_12_-HSL production, however, overproduction of the protein produces a lower level of virulence proteins, thereby RsaL manages homeostasis of the quorum sensing.

Non-coding regulatory RNAs are also involved in quorum sensing suppression. YenS from *Y. enterocolitica* is a non-translated *trans*-RNA (Tsai and Winans, 2011). At low cell densities, the signal-free receptor protein apo-YenR activates the *yenS* transcription binding to a particular sequence on the *yenS* promoter. The YenS base-pairs with 5^′^ region of the signal generator YenI mRNA and then inhibits YenI translation. At high densities, a signal-bound YenR (holo-YenR) cannot do so, resulting in the induction of the quorum sensing. Thus, YenS is a suppressor for the quorum sensing in *Y. enterocolitica.* The photosynthetic soil bacterium *Rhodopseudomonas palustris* produces a non-coding *cis*-RNA that affects the quorum sensing signal receptor expression (Hirakawa et al., 2012). The *cis*-RNA (named asrpaR) is an anti-sense transcript of *rpaR*, a *luxR*-family signal receptor gene. The transcript is induced by the quorum sensing signal *p*-coumaroyl-HSL and the RpaR protein. asrpaR inhibits RpaR translation, presumably by base-pairing with sense transcripts, thus suppressing the quorum sensing activity. Off-targeting technology to disrupt specific target functions utilizing RNA interference with siRNA (small interfering RNA) is undergoing extensive development in the mammalian area, but the major challenge of developing therapeutic applications is currently ongoing. Studies on RNA interference will also be carried out on bacteria.

## CONCLUDING REMARKS AND FUTURE PROSPECTS

Blocking bacterial cell-to-cell communication activity is a novel strategy in antibacterial therapy. In the last 20 years, several approaches to disrupting quorum sensing have been attempted, and these include antagonizing signal sensing, inhibition of signal generation, inactivation of signals, and a variety of agents have been discovered from natural and synthetic libraries. In addition, there are inhibitors like the macrolides where the mode of action has not yet been addressed. The aim of inhibiting quorum sensing is to suppress bacterial virulence and reduce drug resistance/tolerance accompanied with quorum sensing-activated biofilm formation and other innate bio-defense mechanisms by means other than killing bacteria. This strategy is the opposite of bacteriocidal therapies using antibiotics. The benefits might be the suppression of the development of antibiotic resistance, and the ability to expand chemotherapeutic strategies to combat multi-drug resistant (MDR) pathogens. Some of the inhibitors have been evaluated in animal and plant infection models. However, there are many hurdles to overcome for this approach to be used in clinical applications. For example, do these agents only target quorum sensing without any critical and unexpected side effects in addition to their pharmacokinetics (ADME: absorption, distribution, metabolism and excretion)? If they do, can they be administrated with authentic antibiotics to promote healing against multidrug-resistant infections? Recently, a pilot study in clinical trial has been made (Smyth et al., 2010). They used garlic as a quorum sensing inhibitor for 13 CF patients, however, no significant effects were observed compared to placebo group. As they suggested in the preliminary study, reorganization of the study with some modifications (for example, to test in a larger trial) should be necessary. We also need to investigate the potential for selective pressure by quorum sensing inhibition (As we mentioned above, there is a report describing a *mexR* mutation that increases resistance to a furanone inhibitor). Quorum sensing cheater with mutations will be also a critical issue to keep in mind. We will need to answer these questions in the near future to enable us to use these agents as novel antibacterial agents.

## Conflict of Interest Statement

The authors declare that the research was conducted in the absence of any commercial or financial relationships that could be construed as a potential conflict of interest.
